# Molecular Mechanisms of Neuroprotection by Ketone Bodies and Ketogenic Diet in Cerebral Ischemia and Neurodegenerative Diseases

**DOI:** 10.3390/ijms25010124

**Published:** 2023-12-21

**Authors:** Jiwon Jang, Su Rim Kim, Jo Eun Lee, Seoyeon Lee, Hyeong Jig Son, Wonchae Choe, Kyung-Sik Yoon, Sung Soo Kim, Eui-Ju Yeo, Insug Kang

**Affiliations:** 1 Department of Biomedical Sciences, Graduate School, Kyung Hee University, Seoul 02447, Republic of Korea; jwjang0730@naver.com (J.J.); ryfhgnfjdk@naver.com (S.R.K.); jojk4778@gmail.com (J.E.L.); wasdde567777@naver.com (S.L.); thsgudwlr127@naver.com (H.J.S.); wchoe@khu.ac.kr (W.C.); sky9999@khu.ac.kr (K.-S.Y.); sgskim@khu.ac.kr (S.S.K.); 2 Biomedical Science Institute, Kyung Hee University, Seoul 02447, Republic of Korea; 3 Department of Biochemistry and Molecular Biology, School of Medicine, Kyung Hee University, Seoul 02447, Republic of Korea; 4 Department of Biochemistry, College of Medicine, Gachon University, Incheon 21999, Republic of Korea

**Keywords:** ketone bodies, β-hydroxybutyrate, ketogenic diet, mitochondrial dysfunction, oxidative stress, neuroinflammation, cerebral ischemia, neurodegenerative disease, Alzheimer’s disease, Parkinson’s disease

## Abstract

Ketone bodies (KBs), such as acetoacetate and β-hydroxybutyrate, serve as crucial alternative energy sources during glucose deficiency. KBs, generated through ketogenesis in the liver, are metabolized into acetyl-CoA in extrahepatic tissues, entering the tricarboxylic acid cycle and electron transport chain for ATP production. Reduced glucose metabolism and mitochondrial dysfunction correlate with increased neuronal death and brain damage during cerebral ischemia and neurodegeneration. Both KBs and the ketogenic diet (KD) demonstrate neuroprotective effects by orchestrating various cellular processes through metabolic and signaling functions. They enhance mitochondrial function, mitigate oxidative stress and apoptosis, and regulate epigenetic and post-translational modifications of histones and non-histone proteins. Additionally, KBs and KD contribute to reducing neuroinflammation and modulating autophagy, neurotransmission systems, and gut microbiome. This review aims to explore the current understanding of the molecular mechanisms underpinning the neuroprotective effects of KBs and KD against brain damage in cerebral ischemia and neurodegenerative diseases, including Alzheimer’s disease and Parkinson’s disease.

## 1. Introduction

Ketone bodies (KBs) comprise acetone, acetoacetate (AcAc), and β-hydroxybutyrate (BHB). AcAc and BHB serve as crucial alternative energy sources for extrahepatic tissues under various physiological conditions, such as glucose deprivation, fasting, strenuous exercise, the neonatal period, and a low-carbohydrate high-fat ketogenic diet (KD) [1,2]. There are many different types of KDs with varying proportions of macronutrients, which have been well described in recent reviews [3,4]. One representative type of KD is high in fat (70–90% of total calories), moderate protein (7–25%), and low in carbohydrates (3–20%) [3,5]. This high-fat KD (HFKD) is commonly referred to as KD. The very low-calorie KD (VLCKD) is characterized by significant carbohydrate restriction (<50 g/day), adequate protein, and high fat intake, with an average energy intake of 800 kcal/day [4]. Under KD conditions, without or with restriction of calorie, nutritional ketosis is achieved.

In adults, plasma KB concentrations typically range from 0.05–0.1 mM under normal conditions, rising to 1–2 mM after 2 days of fasting and reaching 5–8 mM during prolonged fasting and starvation [1,6,7].

KBs are primarily synthesized in the liver through ketogenesis from acetyl-CoA, produced via fatty acid β-oxidation. The liver can produce up to 300 g of KBs daily, contributing approximately 20% of total energy expenditure during fasting and starvation [2,8,9]. Detailed pathways and regulatory mechanisms of ketogenesis in the liver and ketolysis in extrahepatic tissues have recently been reviewed [2,10].

The human brain, comprising approximately 2% of total body weight, consumes approximately 20% of the total energy expenditure of the body [11]. While glucose is the primary energy source under normal conditions, the brain adapts to KBs during starvation and glucose deprivation. KBs contribute 60–70% of the energy requirements during prolonged fasting [1]. Suckling animal brains exhibit higher circulating KB levels than mature brains, indicating a period of dependence on both KBs and glucose during suckling [12]. Cerebral KB metabolism may play a role in brain development, as the developing brain seems to prioritize KBs for certain metabolic pathways [13]. Brain glial cells, including astrocytes, have been found to produce KBs from fatty acids and ketogenic amino acids via ketogenesis [14,15]. Ketogenic and ketolytic pathways in the brain are briefly described in Section 2.

Although diabetic ketoacidosis is a pathological condition, mild ketonemia resulting from caloric restriction (CR), fasting, vigorous exercise, and KD, has proven beneficial and extends a healthy lifespan [16,17,18,19,20]. Moreover, accumulating evidence has demonstrated that KBs and KD have the potential to treat various diseases such as metabolic diseases including obesity, type II diabetes, and non-alcoholic fatty liver disease, cancer, cardiovascular diseases, and neurological diseases [2,3,10,21,22,23,24]. As the beneficial effects of dietary intervention with KD are mediated through KB production, interest in KBs and KD has been increasing. KBs and KD have been used in the treatment of various neurological diseases, including epilepsy, cerebral ischemia, Alzheimer’s disease (AD), Parkinson’s disease (PD), multiple sclerosis (MS), Huntington’s disease (HD), depression, mood and anxiety disorders, and autism spectrum disorder (ASD) [11,25,26,27,28,29,30,31,32,33,34]. Section 3 describes the latest findings from recent preclinical and clinical studies on the therapeutic potential of KBs and KD in cerebral ischemia and the two most common neurodegenerative diseases, AD and PD.

KBs and KD have been shown to have a variety of beneficial effects on the brain through many different mechanisms [11,35,36]. They exhibit neuroprotective effects by improving mitochondrial function and alleviating oxidative stress. KBs and KD also induce post-translational modifications of epigenetic proteins, reduce neuroinflammation, and affect neurotransmission systems and the gut microbiome [2,9,20,37]. The molecular mechanisms by which KBs exert beneficial effects on health and disease have previously been reviewed with a focus on cancer treatment [10]. In Section 4 of this review, the current knowledge of the molecular mechanisms underlying the neuroprotective effects of KBs and KD is discussed.

## 2. Ketone Body Metabolism in the Brain

### 2.1. Brain KBs Supplied by Ketogenesis in Liver and Astrocytes

The brain comprises two primary cell types: neurons and glial cells. While neurons are central to neurotransmission, glial cells, including astrocytes, oligodendrocytes, and microglia, provide essential physical and chemical support, maintaining an optimal environment for robust neuronal function.

Recent emphasis on the role of KBs alongside glucose as energy sources for brain cells has emerged [38]. Although the liver is the primary supplier of KBs to the brain, astrocytes also contribute to KB synthesis [14,39,40]. In instances such as cerebral ischemia or hypoxia, both hepatic and astrocytic ketogenesis increase, offering neuroprotective effects by enhancing brain energy metabolism and synaptic functions [14,15,41,42]. AMP-activated protein kinase (AMPK), a master energy sensor, plays a crucial role in regulating astrocytic ketogenesis [43], influencing processes like lipid mobilization and KB export.

**Figure 1:**
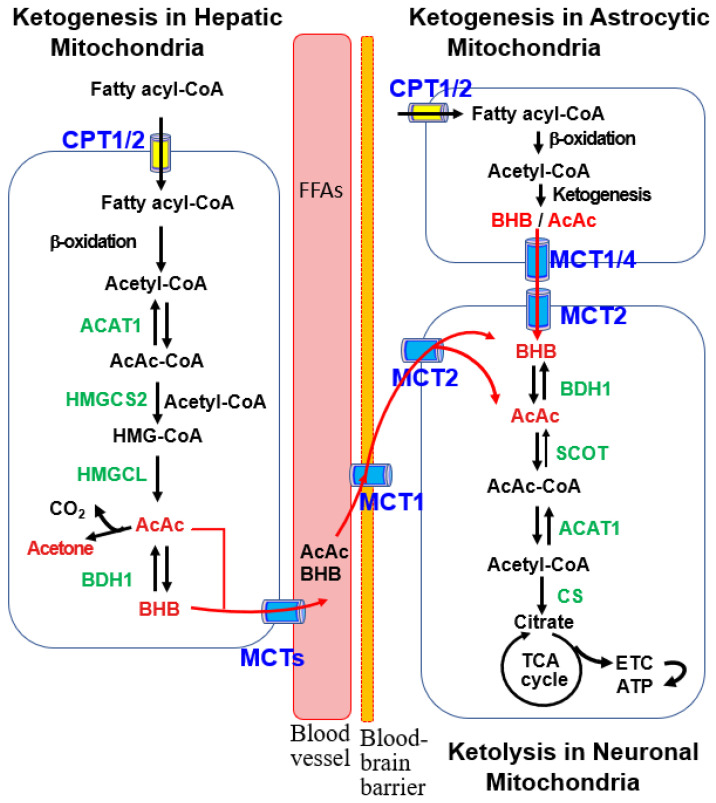
Ketogenic and ketolytic pathways in the liver and brain cells. Ketogenesis primarily occurs in the mitochondria of hepatocytes with acetyl-CoA produced by β-oxidation of fatty acyl-CoA. This process proceeds through stepwise reactions catalyzed by acetoacetyl-CoA thiolase 1 (ACAT1), 3-hydroxy-methylglutaryl-CoA synthase 2 (HMGCS2), 3-hydroxy-methylglutaryl-CoA lyase (HMGCL), and β-hydroxybutyrate dehydrogenase 1 (BDH1). The resulting ketone bodies (KBs), acetoacetate (AcAc) and β-hydroxybutyrate (BHB), are released into circulating blood via monocarboxylic acid transporters (MCTs). After uptake into the brain via MCT1, ketolysis occurs in the mitochondria of brain neurons, where BHB oxidation to acetyl-CoA is catalyzed by BDH1, succinyl-CoA:3-oxoacid-CoA transferase (SCOT), and ACAT1. After conversion of acetyl-CoA to citrate by citrate synthase (CS), ATP is generated through the tricarboxylic acid (TCA) cycle and the electron transport chain (ETC). KBs are also produced in the mitochondria of brain astrocytes, as described in the hepatic ketogenic pathway, and are provided to neurons as an energy source.

The ketogenic pathways in the liver and astrocytes exhibit similarities, as illustrated in Figure 1, which provides detailed insights into hepatic ketogenesis. Acetyl-CoA, generated through fatty acid β-oxidation during low-glucose conditions, initiates ketogenesis in mitochondria [2,10,44]. Carnitine palmitoyl transferase 1/2 (CPT1/2) facilitates the transport of fatty acyl-CoA to the mitochondria. Additionally, ketogenic amino acids, particularly leucine, contribute to acetyl-CoA production for ketogenesis in liver hepatocytes and brain astrocytes [2,35].

The hepatic ketogenesis diagram (Figure 1) illustrates the condensation of two acetyl-CoA molecules, forming acetoacetyl-CoA (AcAc-CoA) catalyzed by acetyl-CoA acetyltransferase 1 (ACAT1) [44]. Subsequently, 3-hydroxy-3-methylglutaryl (HMG)-CoA synthase 2 (HMGCS2) catalyzes the formation of HMG-CoA, the rate-limiting step in ketogenesis. HMG-CoA is further metabolized to AcAc and BHB by HMG-CoA lyase (HMGCL) and BHB dehydrogenase 1 (BDH1) [44]. AcAc and BHB are then transported to the brain through monocarboxylate transporters (MCTs) for ketolysis and ATP production [7,11,35]. As BHB constitutes approximately 70% of the plasma KB pool [7], the terms “KBs” and “BHB” are used interchangeably.

### 2.2. Ketolysis in the Brain

Liver-derived blood KBs, including BHB and AcAc, traverse the blood-brain barrier (BBB) to reach the brain through facilitated diffusion mediated by MCTs [7,11,35]. MCT1 is located at the BBB on microvascular endothelial cells and astrocytes. Astrocytes also express MCT4, while MCT2 is mainly localized in neurons [11,35]. Neuronal BHB transport is also facilitated by sodium-dependent MCT1 (SMCT1).

Within brain neuron mitochondria, BHB, supplied by the liver and astrocytes, undergoes oxidation to AcAc via brain mitochondrial BDH1. Subsequently, AcAc is converted to AcAc-CoA by succinyl-CoA:3-oxoacid-CoA transferase (SCOT), a product of the Oxct1 gene [2,10]. ACAT1 catalyzes AcAc-CoA to generate two acetyl-CoA molecules, entering the tricarboxylic acid (TCA) cycle to produce ATP through the electron transport chain (ETC) (Figure 1).

The extent of KB uptake and ketolysis in the brain is largely influenced by blood KB concentrations [11,45]. MCT1/2 expression regulates the brain’s uptake and utilization of KBs. Previous studies demonstrated increased MCT1/2 expression during ketolysis and after brain injury. Additionally, a KD was found to elevate MCT1 expression and subsequent BHB uptake in the brain [46]. After traumatic brain injury, MCT2 expression is induced in cerebral vasculature, correlating with increased cerebral KB uptake [47]. Studies in mice suggest that KD consumption differentially impacts neuronal and astrocytic gene transcription, influencing mitochondrial function and inflammation, potentially associating with neurodegenerative diseases [48].

Consistent with glucose hypometabolism in the AD brain, glycolytic gene expression is diminished in neurons and oligodendrocytes of patients with AD. Conversely, ketolytic gene expression remains unchanged in neurons, astrocytes, or microglia but shows a significant decrease in oligodendrocytes [49]. Hypometabolism in oligodendrocytes, resulting from impaired glycolytic and ketolytic pathways, is linked to dysmyelination, suggesting potential involvement in AD pathology.

## 3. Therapeutic Potential of KBs and KD in Cerebral Ischemia and Neurodegenerative Diseases

Ketosis induced by KBs and KD intake, ketone ester supplementation, and the administration of medium-chain triglycerides (MCTG) have been utilized in both animal models and humans for treating various neurological diseases, including neurodegenerative diseases. This section outlines recent preclinical and clinical studies on the application of ketogenic therapy for treating cerebral ischemia and two most common neurodegenerative diseases, AD and PD. An overview of clinical studies on the beneficial effects of KD and KBs on neurodegenerative diseases, including AD, PD, MS, and HD is presented in Table 1.

### 3.1. Cerebral Ischemia

Cerebral ischemia, also known as brain ischemia or ischemic stroke, is a detrimental condition where blood flow to the brain is impaired, leading to reduced glucose and oxygen supply and damage or death of brain cells. If left untreated, cerebral ischemia can result in cerebral infarction, hypoxic-ischemic encephalopathy, permanent disability, or death [29,45]. Acute brain injury caused by cerebral ischemia may also induce protein aggregates, increasing the risk of neurodegenerative diseases [65,66].

Several studies have reported the neuroprotective effects of KBs and KD against neuronal damage in various cerebral ischemia animal models [29,45]. BHB pretreatment through infusion or KD intake reduces infarct volume and improves neurological function after focal ischemia in rats and mice [67,68]. Immediate administration of BHB after reperfusion significantly improves ischemia-related neurological scores in mice [69]. Intravenous BHB administration after middle cerebral artery occlusion (MCAO) significantly reduces the infarct area and neurological deficits [42]. Consumption of a KD 3 days prior to stroke induction improves mobility in endothelin-1 model rats, indicating the benefit of KD preconditioning [70]. Similarly, a recent study reported that pre-treatment with KD or post-treatment with BHB improved motor function and reduced infarct volume in a mouse model of cerebral stroke [71]. Moreover, pre-injury KD consumption prevented neurodegeneration due to cardiac arrest-induced cerebral ischemia [72].

The transport and metabolism of KBs are upregulated in both fetuses and neonates [12,45]. It is suggested that KBs and KD may play a role in protecting the brain from damage caused by neonatal hypoxic-ischemic encephalopathy. Overall, these results suggest that KBs and KD may have neuroprotective effects against brain injury caused by cerebral ischemia.

### 3.2. Alzheimer’s Disease

AD, the most common neurodegenerative disease, is characterized by amyloid plaque formation due to amyloid-beta (Aβ) deposition, neurofibrillary tangle formation due to hyperphosphorylation of tau protein, and progressive brain shrinkage due to neuronal loss [3,25]. Although the causes of AD are not yet fully understood, it is proposed that a combination of harmful factors such as age-related changes in the brain, oxidative stress, inflammation, blood vessel damage, and lack of energy supply to the brain are involved. Depending on the stage of the disease, AD patients exhibit a variety of symptoms, including memory loss, decreased learning ability, decreased motor skills, cognitive impairment, and lack of physical control.

A previous study showed that ketogenesis induced by 2-deoxy-D-glucose reduced Aβ deposition and oxidative stress in the 3xTgAD mouse model [73]. Recently, the diverse effects of a KD and KBs have also been reported in different animal models of AD. Diets supplemented with BHB or ketone ester were shown to improve energy utilization and reduce oxidized proteins and lipids, normalizing abnormal behavior in a 3xTgAD mouse model [74,75]. Although KD consumption for 1 month did not affect Aβ levels in a young amyloid precursor protein (APP)/presenilin-1 (PS-1) knock-in AD mouse model, this diet improved motor performance in AD mice [76]. KD consumption for 3 months led to improvements in motor function but not in learning and amyloid or tau deposits in APP/PS-1 and Tg4510 AD mouse models [77]. Another study found that KB administration for 2 months improved learning and memory and lowered oxidative stress in an APP mouse model [78]. Similarly, KD or ketone esters significantly reduced Aβ deposition and phosphorylated tau accumulation, improving Aβ- or tau-dependent pathology and exhibiting anxiolytic and cognition-sparing properties in AD mouse models [79,80].

Clinical studies have shown improvements in verbal memory and cognitive scores in small groups of mild cognitive impairment (MCI) patients who received KD for 6 weeks and in a single-arm study of AD patients with KD administration for 12 weeks [50,57,81]. In a recent randomized crossover trial, 12 weeks of a modified KD significantly improved daily function and quality, but memory improvements were modest [60]. In a pilot clinical study, the modified KD was shown to improve metabolic biomarkers and cerebrospinal fluid Aβ42 [52]. Clinical trials comprising patients with MCI or mild to moderate AD have shown that KD supplementation with ketone ester or MCTG improves short-term cognitive abilities, including memory, language, and attention [51,53,54,56,58,59]. Additionally, an MCTG-based KD improved memory scores and overall cognitive function in patients with mild and moderate AD without significant adverse effects on metabolic parameters [54,55,57,58,82].

Interestingly, an MCTG-based KD reversed cognitive impairment and improved cognitive scores in ApoE4-negative AD patients, but not in ApoE4-positive patients [54,55,59]. These observations suggest that the ApoE4 genotype may play a role in the outcome of KD consumption. Therefore, disease severity, genotype, degree, and duration of KBs and KD may influence treatment outcomes in patients with AD.

### 3.3. Parkinson’s Disease

PD is a neurological disorder characterized by motor deficits resulting from unintentional or uncontrollable movements, including tremors, balance problems, and coordination difficulties [3,25,26]. PD progresses over time, causing significant difficulties in walking and speaking. PD is associated with the loss of dopaminergic neurons in the brain. Although the causes of PD are not yet fully understood, genetic factors, environmental toxins, oxidants, and aging have been suggested as potential causes of dopaminergic neuronal loss in PD.

In a 1-methyl-4-phenyl-1,2,3,6-tetrahydropyridine (MPTP)-induced PD animal model, BHB infusion significantly reduced the loss of dopaminergic neurons and motor deficits [83]. The administration of BHB or KD before MPTP injury improved motor function and reduced dopaminergic neuron loss [84,85]. BHB treatment significantly improved motor dysfunction and dopaminergic neurons in lipopolysaccharide (LPS)-induced PD model rats by inhibiting microglial activation [86]. KD also protected dopaminergic neurons by increasing glutathione levels in PD model rats injected with 6-hydroxydopamine (6-OHDA) [87].

In a pilot clinical study, 8 weeks of KD consumption improved cognitive function but not motor function compared to consumption of a low-fat diet [63]. In a randomized controlled trial, PD patients who consumed KD also showed significant improvement in non-motor symptoms compared to those that consumed a low-fat diet [62]. Additionally, one study found that KD for 28 days improved both motor and non-motor symptoms [61]. These preclinical and clinical observations suggest that KBs and KD may have therapeutic potential in the treatment of PD.

## 4. Molecular Mechanisms of the Neuroprotective Effects of KBs and KD

### 4.1. KBs as an Energy Source for the Brain

Glucose is recognized as the primary energy source for the brain. Consequently, impaired glucose metabolism has been proposed as a contributor to aging and several neurological diseases, including AD and PD [88,89,90]. Blood glucose crosses the BBB through glucose transporters (GLUTs). Impaired glucose transport and metabolism have been observed in some patients with MCI and AD long before symptoms manifest [39,88,89,90]. Deficiencies in GLUT1 and GLUT3 may account for lower glucose uptake in AD brains compared to normal brains.

In contrast to glucose, the utilization of KBs remains unaffected in the brains of elderly individuals and patients with AD. As illustrated in Figure 1, the ketolysis of brain KBs and mitochondrial ATP production through the ETC can supply energy for neurotransmission [17,44]. Therefore, KBs may compensate for the deficiency in brain glucose in MCI and AD, thereby delaying cognitive decline [90,91]. Moreover, in situations where both glucose and KBs are available, certain brain regions preferentially take up KBs, indicating that KBs are the preferred energy source for the brain in certain circumstances [92].

**Figure 2:**
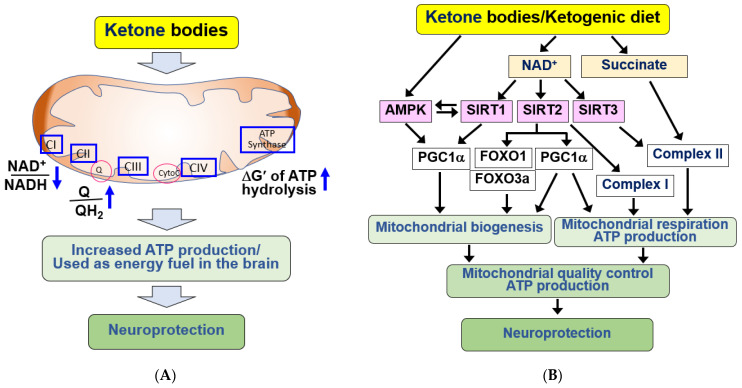
Neuroprotective effects of KBs and KD by providing energy source and improving mitochondrial dysfunction in the brain. (**A**) KBs serve as energy fuel for the brain. KBs promote ATP production by decreasing the NAD^+^/NADH ratio and increasing the coenzyme Q/QH_2_ ratio and ΔG’ of ATP hydrolysis. KBs exert neuroprotective effects through its use as an energy fuel. (**B**) KBs exert beneficial effects by improving mitochondrial function. KBs and KD improve mitochondrial dysfunction by increasing mitochondrial biogenesis through SIRT1/2/3, AMPK, PGC1α, and FOXO1/3a pathways after increasing NAD^+^ production. KBs and KD also increase mitochondrial respiration through PGC1α, complex I/II. The signaling pathways mediated by KBs and KD improve mitochondrial quality control and increase ATP production, resulting in neuroprotection. All arrows indicate activation.

A correlation has been shown between increased blood KB concentration and enhanced memory. Consequently, KB supplementation facilitates the preservation or improvement of brain function and neuronal survival, offering therapeutic potential for the treatment of neurodegenerative diseases [39,82,89,90]. BHB serves as a more efficient energy source than glucose during nutritional deficiencies. BHB reduces the NAD^+^/NADH ratio, oxidizes the coenzyme Q/QH_2_ couples, and increases the ∆G’ of ATP hydrolysis, thereby promoting ATP production [26,93] (Figure 2A). During aging and neurodegenerative diseases, BHB can emerge as a crucial energy source for the brain, mitigating the energy crisis [11,88,94]. Additionally, KB utilization through fasting, ketone esters, and a KD has been shown to enhance brain network stability in young adults, suggesting the potential of KBs in protecting the brain during aging [95]. In summary, the therapeutic applications of KBs and KD are primarily related to their role in energy metabolism.

### 4.2. Beneficial Effects of KBs and KD on Mitochondrial Function

Substantial evidence supports the notion that mitochondrial dysfunctions, including impaired ETC and ATP production, oxidative stress from excessive reactive oxygen species (ROS), mitochondrial DNA damage due to mutations, and defective mitochondrial dynamics and biogenesis, are primary contributors to the pathogenesis of neurodegenerative diseases [39,82,96]. In AD brains, complex IV of the ETC is impaired, whereas in PD brains, complex I activity is compromised. This impaired mitochondrial oxidative phosphorylation reduces cerebral glucose metabolism, leading to neurodegenerative diseases [26,97].

KBs and KD have demonstrated the ability to enhance mitochondrial function in cerebral ischemia and neurodegenerative diseases [26,69,98,99,100,101]. The molecular mechanism underlying the beneficial effects of KBs and KD involves increased NADH oxidation via KB catabolism [39,82]. Elevated NAD^+^ levels play crucial roles, such as stimulating mitochondrial biogenesis and respiration, by serving as a cofactor for NAD^+^-dependent enzymes in cellular signaling processes [102] (Figure 2B).

Sirtuins (SIRTs, class III histone deacetylases, HDACs) and the DNA damage repair enzyme, poly-ADP-ribose polymerase-1 (PARP-1), are representative NAD^+^-dependent enzymes activated by KBs, playing pivotal roles in mitochondrial function [102,103,104]. Brain metabolism by KBs upregulates cytosolic SIRT1/2 by increasing NAD^+^, and SIRT1/2 stimulates mitochondrial biogenesis through peroxisome proliferator activated receptor γ coactivator 1α (PGC1α) [82,105,106,107]. SIRT1 plays a key role in mediating diverse protective effects of KBs in the brain [108]. SIRT1 activates AMPK through deacetylation and activation of the AMPK kinase, liver kinase B1 (LKB1). AMPK also regulates mitochondrial respiration and activates SIRT1 [109]. Therefore, AMPK and SIRT1 activate each other to form a positive feedback loop.

BHB activates AMPK and regulates mitochondrial biogenesis [107]. AMPK and SIRT1 can directly affect PGC1α activity through phosphorylation and deacetylation, respectively [105]. Activation of PGC1α positively regulates mitochondrial dynamics, biogenesis, and oxidative phosphorylation. SIRT1/2 also positively affect mitochondrial ATP production [105,107]. In cortical neurons, D-BHB improves mitochondrial function and stimulates mitochondrial biogenesis, autophagy, and mitophagy by upregulating FOXO1, FOXO3a, and PGC1α, in a SIRT2-dependent manner [107].

BHB-induced NAD^+^ production in brain mitochondria enhances complex I integrity and activity [110,111,112,113]. Under conditions of complex I deficiency caused by mitochondrial (mt) DNA mutations, KB exposure significantly restores the stability and activity of complex I, increasing ATP synthesis. This suggests that KBs promote the repair of complex I damage [111,114]. Mitochondrial SIRT3 can bind to complexes I and II, increasing their activity [103], indicating that SIRT3 activation mediates the effects of BHB on the ETC and ATP production. BHB catabolism generates succinate, the oxidative fuel for succinate dehydrogenase, supplying electrons directly to complex II, bypassing complex I abnormalities, stabilizing the complex, and reducing ROS production [26,67,83,115,116]. Therefore, the neuroprotective effect of BHB is linked to the improvement of mitochondrial complexes I and II and the resulting enhancement of ATP production [69]. Similar to KBs, KD-induced nutritional ketosis increases both mitochondrial respiration and homeostasis, suggesting its therapeutic potential for the treatment of chronic and degenerative diseases with mitochondrial dysfunction [99]. Similar to KBs, KD-induced nutritional ketosis increases both mitochondrial respiration and homeostasis, suggesting its therapeutic potential for the treatment of chronic and degenerative diseases with mitochondrial dysfunction [99].

### 4.3. Anti-Apoptotic and Antioxidant Effects of KBs and KD in the Brain

Apoptotic cell death in brain tissue is a key feature of neurodegeneration, and blocking apoptosis increases neuronal survival, slowing the neurodegenerative process [114]. Oxidative stress is also associated with neurodegenerative progression and neuronal loss through apoptosis. Mitochondria are a major source of ROS generation and play a crucial role in regulating apoptotic cell death [93,97]. The induction of apoptotic cell death is often preceded by the opening of the mitochondrial permeability transition pore (mPTP). This event causes mitochondrial dysfunction, releasing cytochrome C into the cytoplasm, activating caspases and apoptosis [114].

**Figure 3:**
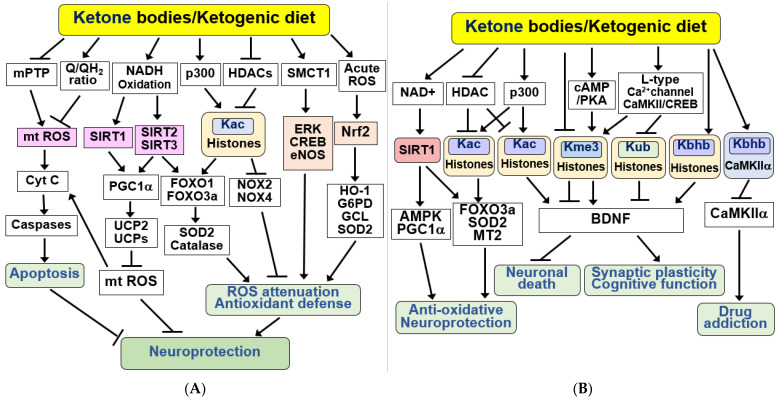
Signaling functions of KBs and KD for anti-apoptosis, antioxidant defense, and epigenetic and post-translational modifications. (**A**) KBs exert beneficial effects by inducing anti-apoptotic and antioxidant defenses. KBs and KD protect neurons from apoptosis by inhibiting the mitochondrial permeability transition pore (mPTP), mt ROS production, and caspase activation. KBs and KD induce antioxidant defenses through the SIRT1/2/3, PGC1α, FOXO1/3a, and ERK/CREB/eNOS pathways and acetylation of histones, resulting in stress resistance and neuroprotection. The antioxidant Nrf2 signaling pathway is activated via acute ROS. (**B**) KBs and KD exhibit their beneficial effects through epigenetic and post-translational modifications of histones and non-histone proteins. KBs exert their neuroprotective effects through the SIRT1/AMPK/PGC1α pathway in a NAD^+^-dependent manner. Additionally, KBs regulate HDAC and p300 to promote acetylation at lysine 27 of histone H3 (H3K27Ac, Kac). KBs increase brain-derived neurotrophic factor (BDNF) expression by inducing acetylation (Kac), increasing trimethylation at lysine 4 (H3K4me3, Kme3), decreasing trimethylation of H3K27 (H3K27me3, Kme3), reducing ubiquitination at lysine 119 of histone H2A (H2AK119ub, Kub), and increasing β-hydroxybutyrylation on lysine 9 of histone H3 (H3K9bhb, Kbhb). KBs also increase the Kbhb of CaMKIIα, thereby reducing CaMKIIα activity and drug addiction. All arrows indicate activation and truncated cut lines indicate inhibition.

KBs and KD have been reported to alleviate oxidative stress and apoptosis in various cell systems and animal models of neurodegenerative diseases. The anti-apoptotic and antioxidant effects of KBs have undergone extensive study in the brain in vitro and in vivo [83,110,117,118,119]. The signaling pathways responsible for these effects are summarized in Figure 3A. Firstly, treatment with KBs affects mitochondrial function by preventing prolonged mPTP opening. Additionally, BHB catabolism increases the coenzyme Q/QH_2_ ratio, reducing mitochondrial ROS generation [93,97]. KBs attenuate apoptotic cell death by reducing mitochondrial ROS production, mPTP opening, and cytochrome C release in neurons isolated from rat brains [114,118]. KBs and KD may protect neurons from brain damage by improving mitochondrial function during acute cerebral ischemia and chronic neurological disorders, including AD and PD [45,83,120].

KBs have been reported to reduce glutamate-induced cell death and free radical formation by increasing NADH oxidation (resulting in elevated NAD^+^ levels) and enhancing mitochondrial respiration in neocortical neurons [110]. BHB and KD were found to activate SIRT1 and rescue premature aging in a mouse model of Cockayne syndrome [121]. SIRT1 activation following status epilepticus in rats has been shown to enhance PGC1α, superoxide dismutase 2 (SOD2), and uncoupling protein 2 (UCP2) [106]. Increased expression and activity of UCPs (UCP2, 4, and 5) were found in the brains of mice fed a KD [104,115]. Increased UCPs can promote mitochondrial respiration and reduce ROS production by uncoupling oxidative phosphorylation and ATP production [115,122].

SIRT3 activation plays an important role in regulating mitochondrial redox homeostasis by increasing NAD^+^ levels and subsequently deacetylating FOXO3a and SOD2 [82,98,119,123,124,125,126]. PGC1α activation also mediates the antioxidant effects of SIRT3 in the brain, preventing dopaminergic neuron death [126]. KBs produced locally by astrocytes can attenuate oxidative stress in spinal cord injury by inhibiting class I HDACs, which regulate the expression of antioxidant genes, including FOXO1/3a and its targets, such as SOD2 and catalase [119]. BHB and KD inhibition of class I HDACs show neuroprotection against oxidative stress by downregulating NADPH oxidase (NOX) 2 and 4 [119]. BHB can also induce antioxidant defense by reducing the cytoplasmic NADP^+^/NADPH ratio [20,26,127]. NADPH is used for the biosynthesis of antioxidants, such as reduced glutathione. A KD improves antioxidant capacity by reducing mitochondrial production of ROS and increasing mitochondrial glutathione synthesis in the hippocampus by upregulating glutamate-cysteine ligase (GCL) [128].

The protective effect of BHB is mediated via the upregulation of the KB transporter, SMCT1, and activation of the extracellular signal-regulated kinase (ERK)/cAMP response element binding protein (CREB)/endothelial nitric oxide synthase (eNOS) pathway [129]. The antioxidant nuclear factor erythroid 2-related factor (Nrf2) signaling pathway is activated by acute ROS. BHB also protects the brain from oxidative stress by promoting Nrf2 transcriptional activity and expression of its target antioxidant genes [130]. Nrf2 activation can reduce several pathogenic processes associated with neurodegenerative diseases by upregulating antioxidant defenses, improving mitochondrial function, and suppressing inflammation [131]. Fasting-induced BHB or BHB supplementation prevents ischemic retinal degeneration by upregulating Nrf2 and its target genes, including glucose-6-phosphate dehydrogenase (G6PD), GCL, and SOD2 [132]. Similarly, KD reduced Aβ deposition and neuronal ferroptosis and improved cognitive function in APP/PS-1 mice through Nrf2 activation [133]. Recently, BHB was reported to alleviate oxidative stress and ferroptosis in dopaminergic neurons in a PD model induced by MPTP [134]. This process likely involves the regulation of the zinc finger protein 36 (ZFP36)/acyl-CoA synthetase long-chain family member 4 (ACSL4) axis.

Taken together, these observations suggest that KBs function as stress response molecules, mediating the beneficial effects of a KD through anti-apoptotic and antioxidant defenses, and enhancing stress resistance and neuroprotection.

### 4.4. KBs and KD as Epigenetic and Post-Translational Modifiers of Histones and Non-Histone Proteins

SIRT1 is associated with the health and longevity effects of CR and mediates the neurological benefits of a KD [26]. SIRT1 induces the FOXO3a-dependent antioxidant genes catalase and SOD2 [108,124] (Figure 3B). Additionally, KD has been shown to induce SIRT1-dependent deacetylation of p53, thereby inhibiting proapoptotic p53 function and protecting neurons from apoptosis [121]. SIRT3 regulates cellular metabolism during fasting, CR, and exercise. Upregulated SIRT3 may mediate the KB-induced improvement in learning and memory in ApoE4 mice by increasing neuronal energy metabolism [135]. Intermittent fasting-induced ketosis increases SIRT3 activity and prevents hyperexcitability and hippocampal synapse dysfunction in APP mutant mice [136]. Consistently, dietary KB supplementation was found to induce SIRT3 expression and prevent seizures and early death in a SIRT3 haploidic AD mouse model [137]. BHB and KD protect against GABAergic (producing γ-aminobutyric acid, GABA) neuronal degeneration and excitotoxicity through SIRT3-mediated mechanisms [97].

Aging is a major risk factor for neurodegenerative diseases, and the epigenetic changes associated with aging can cause neurodegeneration [138]. HDAC inhibitors have shown potential in the prevention and control of cognitive dysfunction, memory deficits, and neuroprotection in AD and PD animal models [139,140]. Epigenetic regulation by KD may extend lifespan and protect against age-related diseases [141,142]. BHB exerts its effects by regulating gene expression and enzyme activity through epigenetic and post-translational modifications, including acetylation, beta-hydroxybutyrylation, and methylation [10,143]. BHB induces histone acetylation through the inhibition of HDACs 1, 3, and 4 (classes I and IIa) and increases the activation of transcriptional target genes, including FOXO3a, SOD2, and metallothionein 2 (MT2), in response to ketogenic states [144]. BHB has been shown to improve Aβ-induced cell viability reduction and downregulation of tyrosine kinase receptor A by inhibiting HDAC1/3 in SH-SY5Y cells [145]. Inhibition of class I and II HDACs by BHB in mice contributes to the life-extending properties of CR [20]. Similarly, administration of BHB to *C. elegans* extends their lifespan, delays Aβ toxicity in AD, and reduces α-synuclein aggregation in PD [16]. This finding indicates that BHB may extend lifespan and prevent age-related neurodegeneration through HDAC inhibition [16,20].

BHB and KD improved hyperbaric oxygen-induced spatial memory impairment through increased histone acetylation [146]. KBs promote hyperacetylation of histones and non-histone proteins by increasing acetyl-CoA, a substrate for p300 histone acetyltransferase (HAT) [10,147]. Recent studies have shown that BHB promotes stroke recovery in rodents by reversing stroke-induced GABA transporter-1 (GAT-1) downregulation and dysfunction through HDAC2/3 inhibition, enhancing neural circuit excitability and functional plasticity [148]. Consistently, BHB has been shown to mediate exercise-stimulated expression of brain-derived neurotrophic factor (BDNF) through HDAC2/3 inhibition and H3 acetylation in the hippocampus [149]. BDNF is a neurotrophic factor that promotes neuronal differentiation and survival, synaptic plasticity, and cognitive function [114].

BHB and KD improve hyperbaric oxygen-induced spatial memory impairment through increased histone acetylation [146]. KBs promote hyperacetylation of histones and non-histone proteins by increasing acetyl-CoA, a substrate for p300 histone acetyltransferase (HAT) [10,147]. Recent studies have shown that BHB promotes stroke recovery in rodents by reversing stroke-induced GABA transporter-1 (GAT-1) downregulation and dysfunction through HDAC2/3 inhibition, enhancing neural circuit excitability and functional plasticity [148]. Consistently, BHB has been shown to mediate exercise-stimulated expression of brain-derived neurotrophic factor (BDNF) through HDAC2/3 inhibition and H3 acetylation in the hippocampus [149]. BDNF is a neurotrophic factor that promotes neuronal differentiation and survival, synaptic plasticity, and cognitive function [114].

In addition to histone acetylation, KD and KBs affect histone methylation as well as DNA methylation [143,150]. The anti-seizure effect of KD is associated with inhibition of DNA methyltransferase (DNMT) through increased adenosine, which causes global DNA hypomethylation [142,150,151]. BHB has been shown to enhance BDNF expression by increasing histone H3 lysine 4 trimethylation (H3K4me3) and decreasing histone H2A lysine 119 ubiquitination (H2AK119ub) at the BDNF promoter in hippocampal neurons [152]. The BHB-induced decrease in H2AK119ub levels depends on L-type Ca^2+^ channels and Ca^2+^-calmodulin-dependent protein kinase II (CaMKII)/CREB signaling, whereas an increase in H3K4me3 levels depends on the activation of cAMP/protein kinase A (PKA) signaling. In addition, BHB promotes BDNF expression through increased acetylation of H3 lysine 27 (H3K27Ac), which is mediated by cAMP/PKA/CREB signaling and decreased trimethylation of H3K27 (H3K27me3) by H3K27me3-specific demethylase [153].

Protein beta-hydroxybutyrylation plays important roles in various cellular processes [154,155]. Xie et al. showed that BHB induces lysine beta-hydroxybutyrylation (Kbhb) on histones and upregulates genes involved in starvation response metabolic pathways [156]. In mouse models of depression, H3K9bhb is reduced, and KD or BHB administration exerts antidepressant effects by increasing H3K9bhb levels and BDNF expression, linking BHB and BDNF via H3K9bhb [157]. A recent study revealed that KD consumption and BHB treatment in rats reduced cocaine-induced reinstatement through beta-hydroxybutyrylation of CaMKIIα in the hippocampus [158]. Beta-hydroxybutyrylation of CaMKIIα inhibited phosphorylation and activity of CaMKIIα. Taken together, these results suggest that BHB and KD protect the brain from neurodegeneration through epigenetic and post-translational modifications of histones and non-histone proteins.

### 4.5. KBs and KD as Anti-Neuroinflammatory Signaling Modulators in the Brain

**Figure 4:**
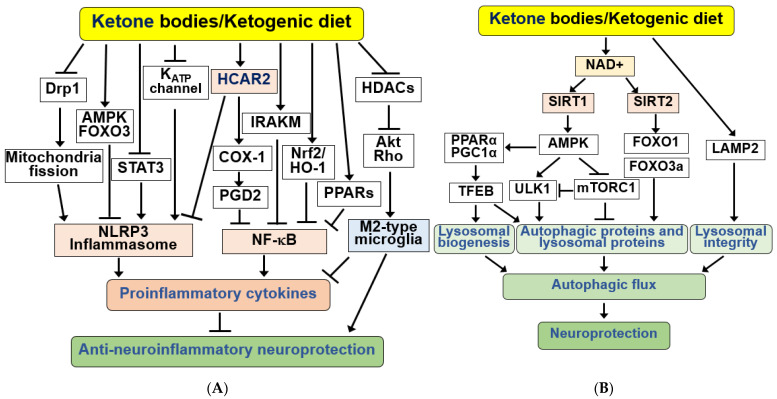
Signaling functions of KBs and KD for anti-neuroinflammation and autophagic flux. (**A**) KBs exert anti-inflammatory responses by activating hydroxycarboxylic acid receptor 2 (HCAR2), which inhibits nuclear factor kappa B (NF-κB) and NOD-like receptor protein 3 (NLRP3) inflammasomes. KBs and KD inhibit the NLRP3 inflammasome through K_ATP_ channels, Drp1, AMPK/FOXO3, and STAT3 pathways, and NF-κB through COX-1/PGD2, IRAKM, PPARs, and Nrf2/HO-1 pathways. KBs and KD also suppress inflammation through Akt/RhoGTPase-mediated ramification via HDAC inhibition and M2 microglial polarization. (**B**) KBs and KD induce autophagy and lysosomal proteins through SIRT1/2, AMPK, ULK1, mTORC1, and FOXO1/3a pathways and maintain lysosomal integrity through LAMP2. These changes increase autophagic flux, resulting in increased neuroprotection. All arrows indicate activation and truncated cut lines indicate inhibition.

Neuroinflammation constitutes a pivotal pathological feature in ischemic stroke and neurodegenerative diseases. The management of neuroinflammation emerges as a crucial strategy to prevent and treat nerve damage in neurological diseases [86,159,160,161]. KBs function as signaling molecules, orchestrating various cellular responses, encompassing inflammatory and immune functions [2,10]. BHB and KD alleviate motor dysfunction and dopaminergic neuron loss in diverse AD and PD animal models by inhibiting microglial activation and the expression of pro-inflammatory cytokines [85,86,162,163]. Similarly, KD pre-treatment or BHB post-treatment enhances motor function and mitigates brain damage by suppressing microglia-mediated proinflammatory responses in a cerebral stroke mouse model [71]. The signaling pathways underlying KBs/KD-induced anti-neuroinflammatory neuroprotection are summarized in Figure 4A.

BHB functions by binding to and activating the hydroxycarboxylic acid receptor 2 (HCAR2), also known as GPR109A, a Gi/o-type G protein-coupled receptor (GPCR) [164]. HCAR2 is required for the neuroprotective effects of BHB and KD in AD, PD, and ischemic stroke models. HCAR2 levels increase in microglia and the substantia nigra under neuroinflammatory conditions in patients with PD [86,104,165]. The anti-inflammatory effects of HCAR2 ligands, such as BHB, nicotinic acid (niacin), and butyrate, have been identified in neurodegenerative diseases [2,161,164,166].

Upon transportation to the brain, BHB binds to HCAR2 on microglial, neutrophil, and monocyte-derived cells, consequently reducing neuroinflammation [161,164]. HCAR2 activation induces a neuroprotective phenotype in macrophages dependent on prostaglandin D2 (PGD2) production by cyclooxygenase 1 (COX1) [160]. The BHB/HCAR2 signaling pathway inhibits inflammatory responses by suppressing nuclear factor-kappa B (NF-κB) activation [86,160,167]. Knockdown of HCAR2 or treatment with pertussis toxin (PTX), an inhibitor of Gi proteins, in microglia abolishes the effects of BHB on NF-κB activation and proinflammatory cytokine release [167]. In the brain, the anti-inflammatory effects of BHB through HCAR2 and NF-κB prove neuroprotective.

In a mouse model of transient MCAO, KD pre-treatment or BHB post-treatment mitigated ischemic brain damage, reduced infarct volume, and suppressed hyperactivation of microglia by inhibiting NF-κB activation through the interleukin-1 (IL-1) receptor-associated kinase M (IRAKM)-dependent pathway [71]. Similarly, recent research showed that KD consumption enhanced cognitive function, reduced amyloid plaques, and lowered proinflammatory cytokine levels in APP/PS-1 mice by activating Nrf2/HO-1 and inhibiting the NF-κB signaling pathway [168]. Additionally, KD activated peroxisome proliferator-activated receptors (PPARs), inhibiting proinflammatory NF-κB activation and COX2 expression in the mouse hippocampus [169,170].

BHB injection inhibits microglial activation, reduces amyloid plaques, and enhances cognitive function in a 5xFAD mouse model [171]. HCAR2 expression was higher in the brains of 5xFAD mice than in wild-type mice. BHB inhibited the expression of pro-inflammatory cytokines, suppressed APP expression, and increased the levels of the Aβ-degrading enzyme, neprilysin, in an HCAR2-dependent manner [171]. Moreover, KD consumption for 4 months in 5xFAD mice improved cognitive function, prevented synaptic and neuronal loss, reduced Aβ deposition, and diminished microgliosis and neuroinflammation [172].

The activation of NOD-like receptor family pyrin domain-containing protein 3 (NLRP3) inflammasome is associated with inflammatory responses. Previously, BHB was demonstrated to inhibit the NLRP3 inflammasome in macrophages and in vivo inflammatory models [173,174]. Consistently, BHB administration reduced amyloid plaques and microgliosis, inhibited NLRP3 inflammasome activation, and curtailed proinflammatory IL-1β release in 5xFAD mice [175]. BHB inhibited endoplasmic reticulum (ER) stress-induced inflammasome formation through AMPK activation and reduced ROS levels through the AMPK-FOXO3 antioxidant signaling pathway [176]. Systemic BHB administration reduces diabetic retinal damage by inhibiting NLRP3 inflammasome and ER stress through retinal HCAR2 activation [173].

KD improves brain ischemia tolerance and inhibits NLRP3 inflammasome activation by preventing dynamin-related protein 1 (Drp1)-mediated mitochondrial fission [177]. BHB administration has also been demonstrated to exert an anxiolytic effect by suppressing NLRP3-induced neuroinflammation in rodent models of post-traumatic stress disorders [178]. Similarly, BHB has been shown to reverse the loss of dopaminergic neurons and glial activation and inhibit microglial pyroptosis by negatively regulating STAT3-mediated NLRP3, resulting in the downregulation of pro-inflammatory cytokines [85].

BHB alleviates the neurodegenerative processes by regulating activated microglia and astrocytes [159,161,179,180]. BHB exhibits anti-inflammatory neuroprotection by inducing anti-inflammatory M2-type macrophage polarization through HDAC inhibition-triggered Akt/RhoGTPase axis [181].

### 4.6. KBs and KD as Regulators of Autophagy

Autophagy, a lysosome-dependent degradation process, is a key mechanism in maintaining cellular homeostasis and survival. Impaired autophagy in the brain results in the accumulation of abnormal protein aggregates and neuronal loss, culminating in neurodegenerative disorders [182]. Therefore, maintaining proper autophagy is essential to prevent accelerated neurodegeneration [108,182].

BHB and KD have been shown to stimulate autophagic flux in glucose-starved cultured neurons and hypoglycemic rat brains [183]. BHB and KD protect against neuronal damage under hypoglycemic and ischemic stroke conditions by improving impaired autophagic flux and reversing reduced monomeric Aβ levels [184,185,186]. The hippocampus and prefrontal cortex respond differentially to changes in autophagy markers and levels of KB utilization upon KD administration, suggesting a potential interconnection between KB metabolism and autophagy [187]. Moreover, the ketogenic enzyme, HMGCS2, promotes the autophagic clearance of APP and tau/phosphorylated tau, mimicked by KB consumption during AD pathophysiology [188,189]. These observations indicate that HMGCS2-induced KB production plays a crucial role in autophagy regulation. Consistently, KD increased the expression of autophagy-related proteins and ameliorated neuronal death in an epilepsy rat model [190]. Collectively, the neuroprotective effects of KBs and KD appear to be linked to their ability to stimulate autophagic flux and correct autophagy defects.

The signaling pathways for neuroprotection by KBs and KD through autophagy regulation are summarized in Figure 4B. BHB has been shown to increase NAD^+^ levels, stimulating lysosomal autophagy by activating SIRT1/2. The SIRT1–AMPK pathway can stimulate neuronal autophagy by regulating several autophagy proteins, including unc-51-like autophagy-activating kinase (ULK1) and mechanistic target of rapamycin (mTORC1). AMPK activates ULK1 by inducing phosphorylation but inhibits mTORC1 [191]. ULK1 increases autophagy by initiating autophagosome formation; however, the mTORC1 signaling pathway inhibits autophagy by inhibiting ULK1 and other autophagic proteins. NAD^+^-dependent SIRT2 activation also increases FOXO1/3a activity, influencing autophagy. The activation and expression of autophagy proteins play a role in preventing neurodegenerative disorders by increasing autophagic flux [108].

Autophagy is enhanced by KBs and KD through the transcription factor EB (TFEB), a key regulator of lysosomal biogenesis [107]. Although the mechanisms by which TFEB is regulated are poorly understood, the recruitment of retinoid X receptor α, PPARα, and PGC1α to the PPAR binding site of the Tfeb gene promoter has been proposed to upregulate TFEB in brain cells [192]. KBs/KD-mediated activation of PPARα and PGC1α and subsequent TFEB induction seem to increase lysosomal proteins and lysosomal biogenesis. D-BHB administration has also been shown to reduce autophagic protein content and cleavage of the lysosome-associated membrane protein 2 (LAMP2) in rat brains injected with N-methyl-D-aspartate (NMDA), thereby increasing lysosomal autophagic degradation and preventing NMDA-induced brain damage [193]. Similarly, D-BHB treatment after MCAO significantly reduced brain damage by preventing LAMP2 cleavage and stimulating autophagic flux in the ischemic core and penumbra [186]. These results suggest that the neuroprotection induced by D-BHB prevents lysosomal rupture, enabling functional autophagy.

### 4.7. KBs and KD as Modulators of Neurotransmission Systems

**Figure 5:**
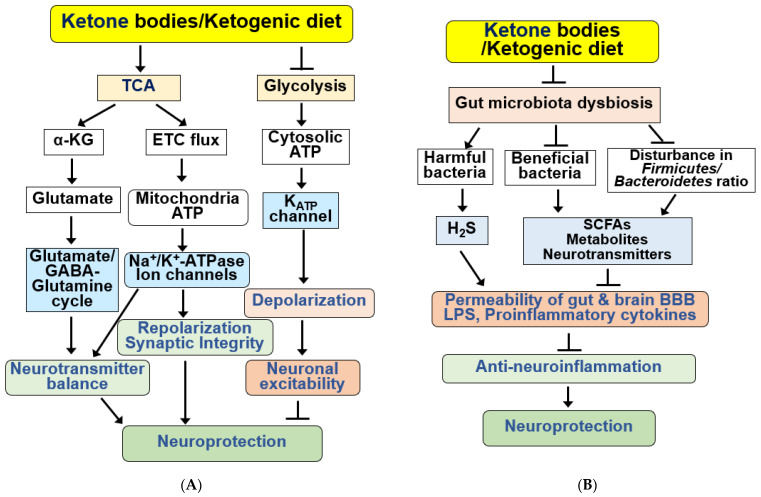
Functions of KBs and KD for the regulation of neurotransmission systems and gut microbiota. (**A**) KBs and KD exert various beneficial effects on the regulation of neurotransmission systems. KBs/KD produce α-ketoglutarate (α-KG) through the TCA cycle and regulate neurotransmitter balance by regulating the glutamate–GABA/glutamate–glutamine cycle. KBs and KD also produce mitochondrial ATP and regulate synaptic integrity through ion channels and Na^+^/K^+^-ATPase. KBs and KD inhibit neuronal excitability through hyperpolarization by inhibiting glycolysis, reducing cytoplasmic ATP levels, and opening K_ATP_ channels. KBs and KD regulate sympathetic function through FFAR3 and N-type Ca^2+^ channels and norepinephrine release. (**B**) KBs and KD improve gut microbiota dysbiosis, increasing beneficial bacteria and reducing harmful proinflammatory bacteria. KB and KD also reverse disturbance in the ratio of *Firmicutes* to *Bacteroidetes*. Bacteria-derived SCFAs, metabolites, neurotransmitters, H_2_S, and LPS regulate the permeability of the gut-brain BBB and the production of proinflammatory cytokines. These alterations by KBs and KD result in anti-neuroinflammatory neuroprotection. All arrows indicate activation and truncated cut lines indicate inhibition.

KBs influence various brain functions and neuronal excitability by regulating neurotransmitter systems [28,194]. The therapeutic impact of KBs on refractory epilepsy may stem from the modulation of neuronal excitability and a decrease in neuronal firing rates [194]. Mitochondrial ketolysis reduces the release of excitatory glutamate, lowering extracellular glutamate levels and elevates inhibitory GABA levels [28]. In cultured neurons, the replacement of glucose with BHB leads to changes in metabolic pathways, enhancing the TCA cycle flux, producing more α-ketoglutarate, and ultimately converting to GABA in GABAergic neurons [38,195] (Figure 5A).

BHB stimulates neurotransmitter recycling in the glutamate–glutamine cycle between neurons and astrocytes [38]. BHB prevents impaired synaptic function, long-term potentiation, and GABAergic activity in hippocampal slices exposed to Aβ and reduces neuronal hyperexcitability in a transgenic APP/PS-1 mouse model [196]. KB-induced mitochondrial ATP production through the TCA cycle and ETC flux can stimulate Na^+^/K^+^-ATPase and play a role in repolarization of the neuronal membrane, thereby regulating neurotransmitter levels [28].

When blood glucose levels are high, active glycolysis increases the cytosolic ATP levels and closes ATP-sensitive K^+^ (K_ATP_) channels in the plasma membrane, depolarizing the membrane. In response to changes in membrane potential, voltage-gated Ca^2+^ channels open, and Ca^2+^ flows into the cell. Ca^2+^ is also released from the ER in response to an initial increase in Ca^2+^ concentration in the cytoplasm. Cytosolic Ca^2+^ concentration is sufficiently high to trigger neurotransmitter release via exocytosis, thereby increasing neuronal excitability. BHB treatment decreases neuronal excitability by inhibiting glycolysis, reducing cytosolic ATP levels, opening KATP channels, causing neuronal membrane hyperpolarization, and decreasing neuronal activity [28,35,197,198]. However, KBs and KD can increase ATP production, and KBs may regulate the opening of KATP channels through various other mechanisms [17,194,197].

BHB treatment reduces NMDA-induced intracellular Ca^2+^ increase in glutamatergic neurons [198]. Although BHB had no direct effect on NMDA-evoked neurotransmitters, BHB was found to increase neurotransmitter release upon stimulation with the KATP channel blocker, glibenclamide, indicating the involvement of K_ATP_ channels in the effects exhibited by BHB [198]. Additionally, KBs have been shown to inhibit glutamate transport by binding to Cl^−^ ions and acting as allosteric modulators of vesicular glutamate transporters [28,194,199].

Interestingly, KD consumption has been shown to improve emotional symptoms, such as anxiety and depression, in patients with AD by modulating the glutamatergic neurotransmission system [32]. Similarly, a KD improves cognitive function in patients with AD and mood disorders in 3xTgAD mice [58,74,79]. Overstimulation of NMDA receptors by glutamate results in neuronal cell death owing to increased Ca^2+^ concentrations and ROS production [32]. The BHB produced by KD ingestion has been shown to reduce the toxicity of amyloid plaques by acting as a glutamate inhibitor at extrasynaptic NMDA receptors. BHB can reduce anxiety and depression in AD pathology by improving the activity of glutamate at the synaptic level with better ATP efficiency and reducing oxidative stress and inflammation [32,195,200].

BHB has been found to bind to free fatty acid receptor 3 (FFAR3, GPR41), a type of GPCR for short-chain fatty acids (SCFAs) that regulates sympathetic nerve activity [201,202]. BHB inhibits voltage-dependent N-type Ca^2+^ channels, and reduces norepinephrine release through FFAR3 activation in rat sympathetic neurons [28,202]. Recently, SCFAs and FFAR3 were reported to enhance cognition and alleviate disease in the 5xFAD mouse model of AD, suggesting that metabolite-sensing FFAR3 may play a protective role against AD [203]. Overall, KBs and KD may enhance neural activity and brain function by regulating neurotransmission.

### 4.8. KBs and KD as Regulators of the Gut Microbiota

The gut microbiome is associated with neurodegenerative diseases by affecting neuroinflammation and metabolic homeostasis [37,204]. Previously, patients with MCI were demonstrated to have increased colonization with pathogenic *Proteobacteria* and *Firmicutes* phyla and a lower abundance of *Bacteroidetes* than cognitively normal subjects [37,204,205]. High levels of harmful bacteria are positively associated with AD biomarkers [205]. Additionally, Gram-negative bacteria containing highly inflammatory LPS are abundant in patients with MCI and AD [205,206]. Similar to AD, disturbances in the gut microbiome (dysbiosis) have been associated with many other neurological disorders, such as PD, epilepsy, ASD, MS, and ischemic stroke [81,204,207,208,209,210].

Gut microbiota have been shown to play an important role in brain function through the gut–brain axis [211,212,213]. The gut microbiota can directly or indirectly influence host metabolism, cognition, and neuroinflammation by producing microbe-derived metabolites, such as SCFAs, lactate, and neurotransmitters, including GABA, dopamine, and serotonin [204,214,215,216].

KD exerts beneficial effects in various neurodegenerative diseases by affecting the diversity and abundance of the gut microbiota and attenuating pro-inflammatory responses [114,204,212,215,217]. A KD alters gut microbiota composition in several neurological diseases [37,204,205,218]. A KD also increases the relative abundance of potentially beneficial gut microorganisms, such as *A. muciniphila* and *Lactobacillus*, and reduces the abundance of harmful proinflammatory bacteria, such as *Desulfovibrio*, *Turicibacter*, and the *Lachnobacterium* genus of the *Firmicutes* phylum [37,205,215,219] (Figure 5B). *Desulfovibrio* produces hydrogen sulfide (H_2_S), which destroys the intestinal mucosal barrier; therefore, reduction in this microorganism by KD may play a role in its beneficial effects [215].

Modified mediterranean KD (MMKD) was shown to improve AD biomarkers, including Aβ and tau protein, in the cerebrospinal fluid of patients with MCI, which correlated with changes in gut microbiome and SCFAs [205]. The abundance of *A. muciniphila* and butyrate significantly increases after MMKD consumption in patients with MCI [205]. Therefore, the effect of KD on the brain is likely mediated by the SCFAs produced by *A. muciniphila* and *Lactobacillus* [219,220]. Additionally, the phylum *Actinobacteria*, family *Bifidobacteriaceae*, and genus *Bifidobacterium* were found to be decreased in the MCI group after MMKD compared to their cognitively normal counterparts, which was consistent with the decrease in lactate and acetate levels in the MCI-MMKD group [205]. Similarly, BHB produced by KD and ketone ester supplementation reduces *Bifidobacteria* and pro-inflammatory Th17 cells in the intestine [221].

KD consumption has been shown to reverse the common autism phenotype of a low proportion of *Firmicutes* to *Bacteroides* species and improve behavioral symptoms in a mouse model of ASD [215,222]. Disturbances in the ratio of *Firmicutes* to *Bacteroidetes* may be associated with gut dysbiosis and negative health outcomes [205]. The ameliorating effect of a KD on ASD-like behaviors is related to reduced levels of pro-inflammatory cytokines and microbiota remodeling [223]. A KD was found to increase the putatively beneficial microbiota, *A. muciniphila* and *Blautia*, and reverse the increase in *Lactobacillus* in the feces of the ASD mouse model. Gut microbial dysbiosis and changes in microbial metabolites have been observed in patients with PD [224]. In patients with PD, non-motor symptoms and gastrointestinal dysfunction often precede motor symptoms [225]. Recent studies revealed that KD and MCTG attenuated impaired motor function, loss of dopaminergic neurons, and inflammation by altering gut microbiota and metabolites in an MPTP-induced mouse model of PD [226,227]. Compared with MPTP-control diet groups, the relative abundance of SCFA-producing bacteria, such as *Blautia* and *Romboutsia*, increased in the KD group with MPTP-MCTG, while the relative abundance of *Desulfomicrobium* decreased significantly [227].

Some studies have reported that KD has negative effects on the gut microbiota [37]. KD has been shown to reduce the overall diversity of the gut microbiome due to its low carbohydrate content [228]. As microorganisms utilize fiber as their primary energy source, some portions of the microbial community cannot survive in KDs with low carbohydrate content. Signs of intestinal dysbiosis were observed in a group of AD rats and patients with MCI after KD consumption [37]. Intermittent fasting reduced hippocampal Aβ deposition, whereas KD worsened gut dysbiosis by increasing *Proteobacteria* and failed to improve memory function in an AD model of rats receiving Aβ(25–35) infusion [229]. A KD also resulted in a further increase in *Enterobacteriaceae* among patients with MCI [205]. Plasma KB levels increase, while plasma and cecal SCFA concentrations decrease in KD [230]. As the neuroprotective effect of a KD exhibited through the intestinal microbial community is influenced by individual factors, such as sex, age, and race, additional studies are needed to identify the underlying mechanism [114].

### 4.9. Adverse Effects of the KD

Although ketosis induced by KBs and KD can provide beneficial effects on various diseases, some adverse effects of KD have been reported. A KD causes a variety of gastrointestinal discomforts, including nausea, vomiting, constipation, diarrhea, low appetite, weight loss, headache, hyperuricemia, and development of dyslipidemia [5,82]. Therefore, more studies, especially long-term studies, are needed to investigate the positive effects, efficacy, and safety regarding the therapeutic application of KBs and KD for neurodegenerative diseases.

## 5. Conclusions

Recently, researchers and clinicians have paid considerable attention to KBs and KD due to their therapeutic potential across various diseases, including metabolic disorders, cancer, cardiovascular diseases, and neurological disorders. Notably, data from both preclinical and clinical trials substantiate the efficacy of KBs and KD in the prevention and treatment of neurodegenerative diseases, such as AD and PD.

**Figure 6:**
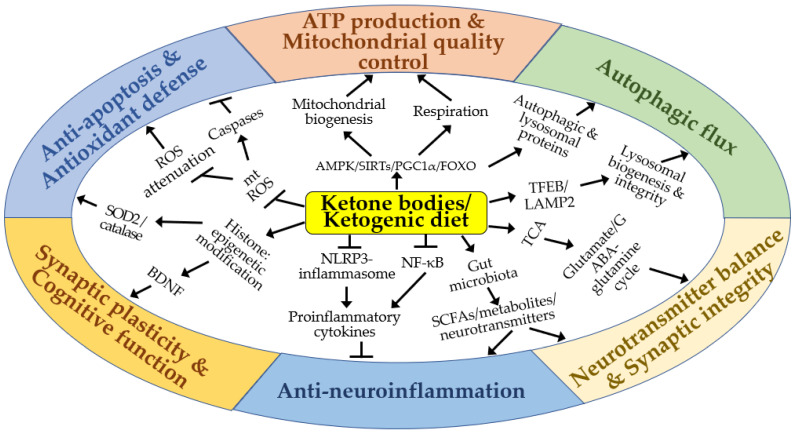
A schematic diagram of the molecular mechanisms by which ketone bodies and ketogenic diet may exert neuroprotective effects in vitro and in vivo. Arrows and truncated lines indicate activation and inhibition, respectively.

KBs and KD confer neuroprotective effects by supplying alternative energy substrates to neurons and enhancing mitochondrial structure and function (Figure 6). Additionally, KBs and KD exhibit positive impacts by reducing oxidative stress and apoptosis, promoting autophagic flux, and regulating gene expression and other cellular functions through epigenetic and post-translational modifications of histones and non-histone proteins. KBs and KD also suppress neuroinflammation and modulate neurotransmitter systems and the gut microbiota. Through these diverse effects, KBs and KD demonstrate significant therapeutic potential for cerebral ischemia and various neurodegenerative diseases.

The molecular mechanisms governing KB levels and the functions of KBs and KD in the brain remain incompletely understood in the context of cerebral ischemia and other neurodegenerative diseases. Consequently, further studies are imperative to elucidate the roles and mechanisms of action of KBs and KD in treating cerebral ischemia and diverse forms of neurodegenerative diseases.

## Figures and Tables

**Table 1:** Overview of clinical studies examining the beneficial effects of the KDs on neurodegenerative diseases.

Disease Population	Number of Participants	Trial Type	Intervention/ Therapy	Duration	Outcome	Reference
MCI	23	RCT parallel	KD	6 weeks	Improved memory function	[50]
MCI	6	RCT parallel	56 g/day MCTG	24 weeks	Improved memory	[51]
MCI	20	RCT cross over	Modified Mediterranean KD	6 weeks	Improved memory performance, brain metabolism	[52]
MCI	83	RCT parallel	15 g twice/day MCTG drink	6 months	Improved episodic memory, language, executive function	[53]
AD, MCI	20	RCT cross over	40 mL MCTG drink	1 dose (single test day	Improved cognitive scores in ApoE4(-) subjects	[54]
AD	152	RCT parallel	MCTG (AC-1202)	90 days	Improved cognitive performance in ApoE4(-) subjects	[55]
AD	1	Single arm	MCTG or ketone ester	20 months	Improved mood, cognitive, and daily activity	[56]
AD	15	Single arm	MCTG supplemented KD	3 months	Improved cognitive scores	[57]
AD	20	Single arm	20 g/day MCTG ketogenic meals	12 weeks	Improved logical memory	[58]
AD	49	RCT cross over	17.3 g/day MCTG	4 weeks	Improved cognition in ApoE4(-) subjects	[59]
AD	26	RCT cross over	KD	12 weeks	Improved daily function, quality of life	[60]
PD	7	Single arm	KD	28 days	Improved motor symptoms	[61]
PD	47	RCT parallel	KD	8 weeks	Improved motor and non-motor symptoms	[62]
PD	14	RCT parallel	Low-carb KD	8 weeks	Improved vocabulary and memory	[63]
MS	60	RCT parallel	KD	3–6 months	Improved quality of life and mental health	[64]
MS	20	Single arm	Modified Atkins diet KD	6 months	Improved fatigue and depression scores	[30]
HD	1	Single arm	Time-restricted KD	48 weeks	Improved motor symptoms, HD-related behavior problems, quality of life	[31]

Abbreviation: AD, Alzheimer’s disease; HD, Huntington’s disease; KD, ketogenic diet; MS, multiple sclerosis; MCI, mild cognitive impairment; MCTG, medium-chain triglyceride; PD, Parkinson’s disease; RCT, randomized controlled trial.

## Data Availability

Not applicable.

## Abbreviations

Aβ, amyloid-beta; AcAc, acetoacetate; ACAT1, acetyl-CoA acetyltransferase 1; ACSL4, acyl-CoA synthetase long-chain family member 4; AD, Alzheimer’s disease; AMPK, AMP-activated protein kinase; APP, amyloid precursor protein; ASD, autism spectrum disorder; BBB, blood-brain barrier; BDH1, β-hydroxybutyrate dehydrogenase 1; BDNF, BHB, β-hydroxy butyrate; CaMKIIα, Ca2^+^-calmodulin-dependent protein kinase IIα; COX-1, cyclooxygenase 1; COX-2, cyclooxygenase 2; CPT1, carnitine palmitoyl transferase 1; CR, calorie restriction; CREB, cAMP response element binding protein; CS, citrate synthase; D-BHB, D-beta-hydroxybutyrate; DNMT1, DNA methyltransferase 1; Drp1, dynamin-related protein 1; eNOS; endothelial nitric oxide synthase; ER, endoplasmic reticulum; ERK1/2, extracellular signal-regulated kinase 1/2; ETC, electron transport chain; FFAR3, free fatty acid receptor 3; FOXO3, forkhead box O3; G6PD, glucose-6-phosphate dehydrogenase; GABA, gamma-aminobutyric acid; GAT-1, GABA transporter-1; GCL, glutamate-cysteine ligase; GLUT1, glucose transporter 1; GPCR, G-protein coupled receptor; HD, Huntington’s disease; H_2_S, hydrogen sulfide; HCAR2, hydroxycarboxylic acid receptor 2; HDAC, histone deacetylase; HMG-CoA, 3-hydroxy-3-methylglutaryl-CoA; HMGCL, 3-hydroxy-3-methylglutaryl-CoA lyase; HMGCS2, 3-hydroxy-3-methylglutaryl-CoA synthase 2; HO-1, heme oxygenase-1; IRAKM, interleukin-1 (IL-1) receptor-associated kinase M; K_ATP_, ATP-sensitive K+; KB, ketone body; Kbhb, lysine beta-hydroxybutyrylation; KD, ketogenic diet; LAMP2, lysosome-associated membrane protein 2; LKB1, liver kinase B1; LPS, lipopolysaccharide; MC, multiple sclerosis; MCAO, middle cerebral artery occlusion; MCI, mild cognitive impairment; MCT, monocarboxylate transporter; MCTG, medium-chain triglyceride; MMKD, modified mediterranean-ketogenic diet; MPTP, 1-methyl-4-phenyl-1,2,3,6-tetrahydropyridine; mPTP, mitochondrial permeability transition pore; MS, multiple sclerosis; mt, mitochondrial; MT2, metallothionein 2; mTOR, mechanistic target of rapamycin; mTORC1, mTOR complex 1; NF-κB, nuclear factor kappa B; NLRP3, NOD-like receptor pyrin domain-containing protein 3; NMDA, N-methyl-D-aspartate; NOX2, NADPH oxidase 2; Nrf2, nuclear factor erythroid 2-related factor 2; 6-OHDA, 6-hydroxydopamine; Oxct1, 3-oxoacid-CoA transferase 1; PARP-1, poly-ADP-ribose polymerase-1; PD, Parkinson’s disease; PGC-1α, peroxisome proliferator activated receptor γ coactivator 1α; PGD2, prostaglandin D2; PKA, protein kinase A; PPARs, peroxisome proliferator activated receptors; PS-1, presenilin-1; ROS reactive oxygen species; SCFA, short-chain fatty acid; SCOT, succinyl-CoA:3-oxoacid-CoA transferase; SIRT, sirtuin; SMCT1, sodium-dependent MCT1; SOD2, superoxide dismutase 2; STAT3, signal transducer and activator of transcription 3; TCA, tricarboxylic acid; TFEB, transcription factor EB; UCP2, uncoupling protein 2; ULK1, unc-51 like kinase 1; ZEP36, zinc finger protein 36.
